# Historical Changes in Self-Rated Health, Memory, and Depressive Symptoms Among Immigrants in the United States

**DOI:** 10.1093/geroni/igaf122.1866

**Published:** 2025-12-31

**Authors:** Yesenia Cruz-Carrillo, Frank Infurna

**Affiliations:** Arizona State University, Tempe, Arizona, United States; Arizona State University, Tempe, Arizona, United States

## Abstract

Empirical evidence has documented historical declines in midlife health, well-being, and cognition, whereas historical improvements have been observed among older adults in the U.S. Less is known about whether recent cohorts of immigrants are doing better or worse than their earlier-born peers and how this relates to broader trends that have been transpiring in the U.S. We examined whether self-rated health, depressive symptoms, and memory have changed across cohorts of middle-aged and older adult immigrants living in the U.S. We also explored the moderating role of gender, race, education, and age at immigration. Multilevel models were applied to longitudinal data from the Health and Retirement Study to examine historical changes in trajectories of self-rated health, episodic memory, and depressive symptoms among immigrants (N = 5,131, 57% women, birth year: M = 1945, range: 1890 to 1973) in midlife and old age. Historical improvements in self-rated health, historical stability in episodic memory, and historical decreases in depressive symptoms were observed across birth cohorts of immigrants in midlife and old age. Race and education moderated historical change. For example, attaining more years of education was associated with less strong historical decreases in depressive symptoms among successive cohorts. Our findings contrast the broader trends observed in the U.S. population; successive cohorts of immigrants demonstrated improvements in each of the included outcomes. Our discussion focuses on potential explanations for these trends, such as migration selectivity, the consideration of the historical context in our understanding of immigrant health, and the protective effects of cultural assets.

